# Transparency as the foundation of discovery

**DOI:** 10.1113/EP093801

**Published:** 2026-06-10

**Authors:** Peter Rasmussen, Damian Miles Bailey, Alex Stewart

**Affiliations:** ^1^ Genmab AS Copenhagen Denmark; ^2^ University of South Wales Pontypridd UK; ^3^ The Physiological Society London UK

**Keywords:** confirmatory inference, exploratory physiology, preregistration, reproducibility, statistical analysis plan

1

We appreciate the correspondence by Richter et al. (2026) stating that rigid analysis plans might limit exploratory work in physiology, and we acknowledge that exploration is key to innovation. Their letter was written in response to the recent editorial by Christensen et al. (2025), which argued that prespecified statistical analysis plans can strengthen methodological rigour and reproducibility in physiological research. Richter and colleagues (2026) interpret this proposal as potentially constraining exploratory physiology and raise concerns about the imposition of clinical trial‐style regulatory frameworks on basic experimental science. However, it is important not to equate all non‐randomised controlled trial physiology research with exploration; doing so conflates the size of a study with its purpose. Regardless of scale or setting (human, animal or laboratory), all studies are governed by the same principles of inference. The essential distinction lies in the reporting; the language used must align with the study type, meaning that confirmatory wording is inappropriate when the analyses are exploratory. At its core, the issue is not whether research is exploratory, but whether the inferential language used accurately reflects the degree of analytical constraint under which findings were generated.

A study is confirmatory when it tests a prespecified hypothesis under analytical constraints fixed before the data were observed. Confirmatory inference requires temporal priority and constrained analytical flexibility. Key elements, such as primary outcomes, statistical models, inclusion criteria and inferential thresholds, must be determined independently of the data used to test them. Only in such conditions do nominal error rates retain their intended meaning. The validity of nominal error rates is a mathematical property of prespecified analytical conditions, not a cultural preference or editorial convention (Neyman & Pearson, 1933). In contrast, exploratory studies retain analytical flexibility. They may be driven by theory and guided by expectations, but their analyses are not fully constrained in advance. Their findings are therefore appropriately interpreted as hypothesis generating or provisional rather than as formal tests conducted under controlled error rates. Crucially, this distinction is orthogonal to context. It applies equally to large pharmaceutical trials, small human studies, mechanistic animal experiments and laboratory investigations. When confirmatory and exploratory research are not distinguished clearly, the inferential boundary between them risks becoming blurred. Investigators might conduct work motivated by specific theoretical expectations and make confirmatory‐style claims, yet without analytical commitments that preceded data inspection. In such cases, the evidential weight implied by statistical inference might exceed what the design justifies, not because of misconduct, but because analytical flexibility and hypothesis testing have not been separated clearly. This concern reflects universal features of human cognition and decision‐making under uncertainty rather than any presumption of ill intent. Modern data analysis inevitably introduces multiple researcher degrees of freedom, including outcome selection, model specification and subgroup definition, each of which can subtly influence inference when not documented ‘transparently’ (Gelman & Loken, 2014).

We recognize that much physiology research is genuinely exploratory, and we do not seek to constrain such work. However, tension arises when exploratory studies are written and interpreted using confirmatory language. If an investigation is conducted with analytical flexibility and without prespecified inferential commitments, its findings should be framed accordingly. Conversely, if authors wish to present results as formal tests that ‘demonstrate’, ‘establish’ or ‘provide evidence for’ specific hypotheses, then prespecification of key elements of the design and analysis must be accepted as part of that inferential claim. One cannot simultaneously retain analytical flexibility and claim the evidential weight of constrained hypothesis testing. To that end, preregistration does not freeze scientific thinking, prohibit adaptive inquiry or prevent investigators from pursuing unexpected findings; it simply distinguishes which analytical decisions preceded data inspection and which followed it (Nosek et al., 2018).

The responsibility for this alignment does not rest solely with authors. Editors, reviewers and readers routinely expect clear hypotheses and definitive conclusions. Manuscripts described as exploratory often face pressure to identify ‘main’ findings and to frame them in confirmatory terms, and they may struggle to be published in high‐impact journals without such framing. Preregistration provides structural support for clearly labelled exploratory analyses without penalizing discovery. By distinguishing planned analyses from *post hoc* ones, it reduces the incentive retrospectively to elevate exploratory findings to confirmatory status. Registered Reports further reduce incentive to frame findings around statistical significance by decoupling publication decisions from outcome direction or magnitude.

Richter et al. (2026) note that preregistration might be particularly valuable for early‐career investigators, a point we fully endorse. It is equally relevant for senior‐led studies. Statistical analyses are often conducted by specific members of the author team, reflecting a division of expertise rather than independent replication across all authors. Although all authors remain accountable for the integrity of the work, formal internal validation of analyses is not always reported. Across the broader physiology literature, explicit disclosure of a prespecified statistical analysis plan, even in brief form within an approved protocol, is still relatively uncommon, and formal internal validation of analyses is not always reported. This can create a structural tension, in which analytical flexibility is retained while findings are, nevertheless, framed using confirmatory language. In such situations, there is a natural tendency for studies to carry the appearance of ‘constrained’ hypothesis testing even when the underlying analytical pathway remained flexible. Importantly, preregistration is not designed to prevent fabrication or fraud, but to reduce undisclosed analytical flexibility, a distinct and well‐characterised contributor to irreproducibility. The fact that concerns about the reliability, reporting and reproducibility of clinical research persist only further highlights the importance of structural transparency in the scientific process (Goldacre et al., 2019; Ioannidis, 2005).

Preregistration offers a straightforward safeguard by aligning analytical commitments with the strength of the claims made. These considerations are not matters of trust but of structure. Inferential statistics carry their advertised meaning only when analytical choices are not conditioned on the data used to evaluate them. Flexibility is essential in exploratory work, but so is transparency. Clearly distinguishing prespecified analyses from data‐driven ones allows exploratory findings to retain their proper epistemic status without being interpreted as confirmatory tests. In high‐dimensional ‘omics’ settings, where multiplicity and analytical branching are substantial, it is arguable that transparent separation of discovery from validation is even more important. In this sense, analytical transparency functions as inferential infrastructure, akin to documenting experimental conditions or instrument calibration.

From the perspective of *Experimental Physiology*, this alignment can be achieved proportionately through preregistration. Authors who wish to make confirmatory claims should, at a minimum, disclose via research protocol registration the prospectively specified key analytical elements within an approved protocol, thereby showing that key outcomes and analytical decisions were defined before data inspection. This is not a call for broader ethical disclosure requirements; rather, it reflects the importance of transparently documenting prespecified analytical elements within existing approved protocols. In the absence of such disclosure, confirmatory language should be moderated to reflect the exploratory nature of the work. The aim is not to introduce administrative barriers, but to ensure that inferential claims are commensurate with design. We are committed to making preregistration practical and proportionate, while recognising the genuine resource constraints under which many physiology laboratories operate. Implementation should therefore be flexible and proportionate to study scale, complexity and available resources, rather than imposed through a one‐size‐fits‐all model.

At the same time, we remain committed to the (perhaps idealistic but evidence‐informed) conviction that transparency strengthens science. By reducing selective reporting and *post hoc* reinterpretation, preregistration can streamline review and support more efficient and transparent publication. Additionally, in *Experimental Physiology*, preregistration is linked to in‐principle acceptance, ensuring publication following successful completion of the study, a long‐term benefit that should far outweigh the perceived administrative burden of disclosing a protocol. In doing so, it can simplify the path to dissemination. If widely adopted across physiology journals, it would shorten the road to publication, reduce iterative cycles of review focused on narrative framing, and make the process both more efficient and more transparent for investigators.

Our position is not that all physiology must be preregistered, nor that exploratory research should be constrained. Rather, when confirmatory inference is claimed, transparency about what was specified before data were observed is essential to preserving the epistemic integrity of statistical evidence. Exploration, too, benefits from transparency, because it ensures that discovery is not confused with formal testing. Exploration should flourish, particularly in high‐dimensional omics research and mechanistic probing, where discovery is intrinsic and analytical multiplicity unavoidable. But inferential claims must match design. Transparent preregistration, protocol documentation, and explicit labelling of confirmatory versus exploratory analyses preserve both creativity and rigour. History shows that many transformative discoveries were both exploratory in spirit and rigorous in documentation; discovery and structure are not opposing virtues. Our aim is not enforcement but clarity, to preserve exploratory creativity while ensuring that evidential claims ultimately remain commensurate with design.

In this spirit, we view the exchange prompted by Christensen et al. (2025) and Richter et al. (2026) not as a ‘disagreement’ about the value of discovery, but as an ‘opportunity’ to refine how physiology communicates its evidence. Exploratory and confirmatory approaches serve complementary roles in scientific progress; one generates new ideas, and the other tests them under conditions that support reliable inference. By making analytical commitments transparent and clearly distinguishing discovery from confirmation, our field can preserve the creative freedom that drives physiology forwards while strengthening the credibility and reproducibility of the knowledge it produces. Transparency, therefore, should not be seen as a constraint on discovery, but as the very foundation upon which durable discoveries are built.

## AUTHOR CONTRIBUTIONS

Peter Rasmussen conceptualised the work. Peter Rasmussen, Damian Miles Bailey and Alex Stewart drafted and revised the manuscript critically. Peter Rasmussen, Damian Miles Bailey and Alex Stewart approved the final version of the manuscript and agree to be accountable for all aspects of the work in ensuring that questions related to the accuracy or integrity of any part of the work are appropriately investigated and resolved. All persons designated as authors qualify for authorship, and all those who qualify for authorship are listed.

## CONFLICT OF INTEREST

The authors declare no conflicts of interest.

## FUNDING INFORMATION

None.
